# Anoxic cell rupture of *Prevotella bryantii* by high-pressure homogenization protects the Na^+^-translocating NADH:quinone oxidoreductase from oxidative damage

**DOI:** 10.1007/s00203-019-01805-x

**Published:** 2020-01-18

**Authors:** Lena Schleicher, Günter Fritz, Jana Seifert, Julia Steuber

**Affiliations:** 1 Institute of Microbiology, University of Hohenheim, Stuttgart, Germany; 2 Institute of Animal Science, University of Hohenheim, Stuttgart, Germany

**Keywords:** EmulsiFlex-C3 homogenizer, *Prevotella bryantii*, Cell rupture, Na^+^-translocating NADH:quinone oxidoreductase (NQR), Superoxide

## Abstract

Respiratory NADH oxidation in the rumen bacterium *Prevotella bryantii* is catalyzed by the Na^+^-translocating NADH:quinone oxidoreductase (NQR). A method for cell disruption and membrane isolation of *P. bryantii* under anoxic conditions using the EmulisFlex-C3 homogenizer is described. We compared NQR activity and protein yield after oxic and anoxic cell disruption by the EmulsiFlex, by ultrasonication, and by glass beads treatment. With an overall membrane protein yield of 50 mg L^–1^ culture and a NADH oxidation activity of 0.8 µmol min^−1^ mg^−1^, the EmulsiFlex was the most efficient method. Anoxic preparation yielded fourfold higher NQR activity compared to oxic preparation. *P. bryantii* lacks genes coding for superoxide dismutases and cell extracts do not exhibit superoxide dismutase activity. We propose that inactivation of NQR during oxic cell rupture is caused by superoxide, which accumulates in *P. bryantii* extracts exposed to air. Anoxic cell rupture is indispensable for the preparation of redox-active proteins and enzymes such as NQR from *P. bryantii*.

**Electronic supplementary material:**

The online version of this article (10.1007/s00203-019-01805-x) contains supplementary material, which is available to authorized users.

*Prevotella bryantii* is a Gram-negative obligate anaerobe, which is found in anoxic zones of the intestine, such as the rumen of cows (Deusch and Seifert 2015). During the fermentation of sugar (Hackmann et al. 2017), NADH oxidation is catalyzed by a single enzyme, the Na^+^-NADH:quinone oxidoreductase (NQR) (Deusch et al. 2019). The NQR sodium pump has been mainly studied in facultative anaerobes, such as *Vibrio cholerae* (Steuber et al. 2015). In *V. cholerae*, respiration in the presence of O_2_ led to the formation of superoxide (Muras et al. 2016), which inactivates the NQR (Pfenninger-Li et al. 1996; Steuber et al. 1997), due to modifications of its redox cofactors (flavins and iron-sulphur centers) (Macomber and Imlay 2009). In aerobes, this is prevented by protective enzymes such as superoxide dismutase (SOD). Here, we addressed the putative inactivation of *P. bryantii* NQR by oxidative damage by comparing its activity in oxically and anoxically prepared membranes. We also compared overall yield of membrane proteins after cell rupture by the EmulsiFlex, by ultrasonication, or by treatment with glass beads. *P. bryantii* NQR is prone to oxidative damage during preparation of cellular extracts since *P. bryantii* lacks SOD, as confirmed by enzymatic tests.

The very first and most critical step in the purification of proteins is the breakage of cells. Different methods can be used to disrupt microorganisms, like enzymatic lysis by destabilizing the cell wall. Alternatively, ultrasonication or compulsion followed by rapid relieve of pressure can be performed. The latter is achieved with the help of the EmulsiFlex-C3 homogenizer (Avestin Inc., Ottawa, Canada) (Tong 2011). Unlike lysis by enzymes or by ultrasonication, which can be performed in an anaerobic chamber under exclusion of O_2_, high-pressure cell rupture under anoxic conditions, required for preparation of oxygen-labile components of cells, is difficult to achieve. We established a method for anoxic rupture of *Prevotella bryantii* B_1_4 using the EmulsiFlex-C3 homogenizer. The protocol for anoxic cell disruption involves the use of an anaerobic chamber (COY laboratory products) and special equipment (supplementary data, supplementary Fig. S1). Buffers were made anoxic by flushing with N_2_ before entering the chamber. Outside of the chamber, the anoxic suspension was handled in gas-tight vials or syringes. Cells were cultivated in anoxic medium supplemented with the reducing agent L-cystein HCl (1 g L^–1^) and resazurin (0.5 µM, added from a 0.5 mM stock solution in H_2_O). Resazurin is a redox indicator, which turns pink if O_2_ is present, but remains colorless if anoxic and reducing conditions are maintained during handling of cell suspensions (Uchino 2013). The continuous, optical inspection of media and cellular extracts is important. If cell suspensions and cellular extracts turn pink, the overall procedure is not anoxic any longer, and samples must be discarded. It is not recommended to re-reduce the solutions by adding L-cystein HCl. For harvesting the cells, the culture (OD at 600 nm, 2.5–3.0) grown in gas-tight serum bottles (1 L) was transferred into the anaerobic chamber and filled into gas-tight beakers for subsequent centrifugation outside of the anaerobic chamber (9000 g, 30 min, 4 °C). Afterwards, beakers were placed into the anaerobic chamber, and cells were resuspended twice in anoxic cell lysis buffer (20 mM Tris-H_2_SO_4_ pH 7.5 with 50 mM K_2_SO_4_). 10 g of cells (wet weight) was resuspended in 30 mL 20 mM Tris–H_2_SO_4_ pH 7.5, containing 50 mM K_2_SO_4_, 5 mM MgSO_4_, 1 mM dithiothreitol, 1 mM phenylmethylsulfonylfluorid, 0.1 mM diisopropyl fluorophosphate and traces of DNase I (Roche) (Deusch et al. 2019). A detailed, step-by-step protocol, which is a modification of the protocol described by the manufacturer (https://www.avestin.com/emulsiflex-c3.htm) and by (Tong 2011), is given in the electronic supplementary material (Fig. S1). Importantly, the EmulsiFlex must be modified with a plug, which replaces the screw cap of the funnel (Fig. 1). In this plug three plastic disposable syringes are inserted (without stamp) and closed with rubber plugs. A nitrogen gas bottle is connected to one syringe with a cannula to flush the funnel continuously with N_2_. Continuous flow of N_2_ bubbles is monitored with the help of a tubing ending in a beaker filled with water. One syringe is used to inject anoxic buffer and cell suspensions into the funnel. To disrupt the cells, the outlet tubing is connected to a cannula, which in turn is connected to a syringe in the plug on top of the funnel. In this way, the cells run in cycles through the device and the pressure can be increased to 20.000 psi for 10 min. Afterwards, the outlet tubing is connected with a cannula to a gas-tight serum bottle (Fig. S1). To operate the device oxically again, one simply has to remove the plug.

**Fig. 1:**
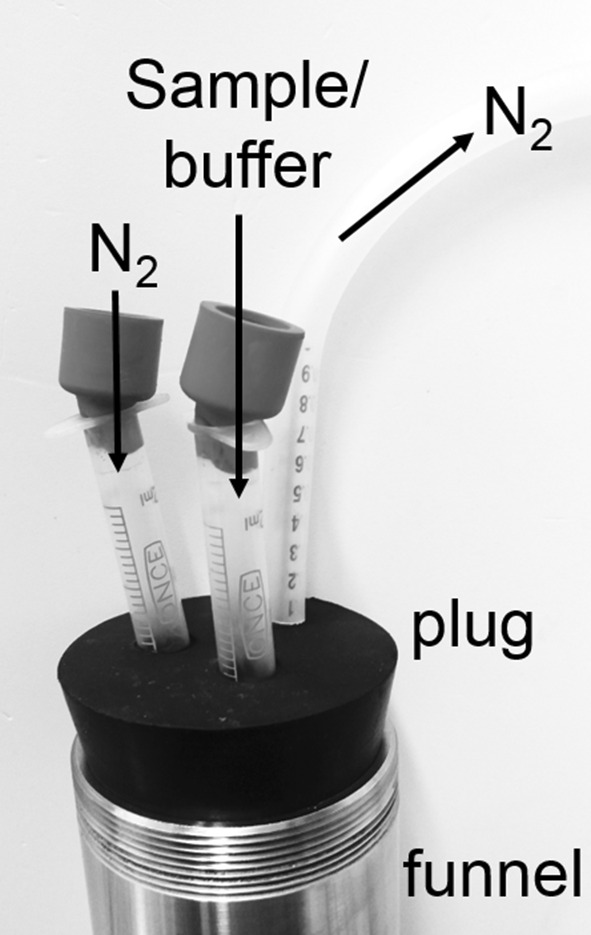
EmulsiFlex-C3 funnel for anoxic cell disruption. The screw cap is removed and replaced by a plug, which containes three plastic disposable syringes. The stamps of the syringes are removed and rubber plugs are inserted on top instead. One syringe is connected to a nitrogen bottle, the other syringe is required for the outlet of N_2_. The third syringe is used to inject anoxic buffer or sample into the system

The method described above was compared to methods based on glass beads (Taskova et al. 2006) or ultrasonication with a sonotrode (Sonopuls 3100 MS 73 Bandelin; 4 min ultrasonication with 10.5 s pulse and 20 s resting time alternately; 75% amplitude; 2.9 kJ). For oxic or anoxic cell rupture by ultrasonication, the sonotrode was inserted into resuspended cells exposed to air, or flushed with N_2_, respectively. For glass bead cell disruption, the cells were resuspended in a 50 mL falcon tube and glass beads (Ø 5 mm) were added. Cells were disrupted by vortexing for 10 min. For anoxic cell disruption with glass beads, this procedure was done in the anaerobic chamber. Membranes were obtained from cell extracts by ultracentrifugation as described previously (Deusch et al. 2019). In subsequent steps, anoxically prepared cell fractions and membranes were never exposed to air, but always manipulated in the anaerobic chamber.

NQR activity with oxically or anoxically prepared membranes of *P. bryantii* (80–100 µg protein) was tested by monitoring the NADH oxidation at 340 nm photometrically, with 100 µM NADH and 100 µM ubiquinone-1 in 20 mM potassium phosphate buffer with 200 mM NaCl at pH 7.5 (Juárez et al. 2009). This experiment was repeated three times for oxically and anoxically prepared membranes (Table 1). Table 1 shows the overall membrane protein yield [determined by the BCA method (Smith et al. 1985)] and the specific NADH oxidation activity of membranes from oxic or anoxic crude extracts from 5 g (wet weight) *P. bryantii* cells. The EmulsiFlex is the most efficient method to break up *P. bryantii* cells. The protein yield is 40–50 mg, which is more than 10 times higher than the protein yield with ultrasonication or glass beads (2–3 mg). In oxic crude extracts, the specific NADH oxidation activity is always similar (0.15–0.22 µmol min^−1^ mg^−1^), independent of the disruption method. In contrast, anoxic cell disruption with the EmulsiFlex yields a fourfold higher activity (0.8 µmol min^−1^ mg^−1^) compared to oxic cell rupture. Anoxic cell disruption with ultrasonication results in a twofold higher activity of extracts (0.46 µmol min^−1^ mg^−1^), compared to the oxically prepared extracts. Anoxic preparation does not result in increased activities if cells are broken with glass beads. These results demonstrate that cell disruption with the EmulsiFlex-C3 homogenizator is the most efficient method for *P. bryantii* cell rupture with respect to NQR activity and overall membrane protein yield. Moreover, up-scaling of the process is easy, since the EmulsiFlex is a continuously operated disruption system where cell suspensions can be loaded repeatedly into the sample cylinder. Up-scaling is easy both under oxic or anoxic conditions, where constant flushing with N_2_ is possible. In contrast, cell rupture by e.g. French Press has a fixed maximum volume of cell suspension. For anoxic rupture with the French Press all suspensions must be transferred into the pressure cell in the anaerobic chamber, preventing the continuous up-scaling of the process.

**Table 1:** Comparison of protein yield and specific NADH oxidation activity of *P. bryantii* membranes. Cells were disrupted with the EmulsiFlex-C3 homogenizer, ultrasonication or glass beads, and under oxic or anoxic conditions, respectively

	Cell rupture method					
	EmulsiFlex-C3 homogenizer		Ultrasonication		Glass beads	
	+ O_2_	-O_2_	+ O_2_	−O_2_	+ O_2_	−O_2_
Protein yield membrane [mg] from 5 g cells (wet weight)	50	40	3	3	2	3
Specific activity of NADH oxidation [µmol min^−1^ mg^−1^] of membranes	0.20 ± 0.02	0.80 ± 0.03	0.22 ± 0.01	0.46 ± 0.02	0.15 ± 0.01	0.21 ± 0.02

We considered that diminished NQR activity in oxically prepared membranes is caused by the inactivation of the enzyme by superoxide. In many organisms, superoxide is removed with the help of superoxide dismutases. For example, *V. cholerae* possesses three SODs (accession numbers UNIPROT: A0A0H3AKF7, A0A0H3AJ73, A0A0H3AIV1) protecting the organism from superoxide. A search for SOD homologs using the UNIPROT and KEGG databases in the *P. bryantii* genome (https://www.ncbi.nlm.nih.gov/Taxonomy/Browser/wwwtax.cgi?lvl=0&id=752555) was not successful. To confirm the absence of SOD in *P. bryantii*, we conducted SOD enzyme activity measurements with a superoxide dismutase activity assay kit (BioVision). Here, xanthine oxidase is used to produce superoxide anions. With these superoxide anions a water-soluble formazan dye is formed, which absorbs at 450 nm. This reaction is prevented by SODs. The kit is designed for an endpoint measurement with a microplate reader. We used a 0.5 cm quartz cuvette and a UV/VIS spectrophotometer (SPECORD® S600 AnalytikJena) to follow the absorbance at 450 nm over time. Reaction is started by adding 50 µl of the enzyme working solution, containing the xanthine oxidase (Fig. 2a). By adding 100 U SOD (from bovine, Sigma) the formation of formazan is prevented completely (Fig. 2b). If *P. bryantii* crude extract (0.13 mg protein) is added, no inhibition of formazan production is observed (Fig. 2c). We also tested *P. bivia* crude extracts for SOD activity. Unlike *P. bryantii*, *P. bivia* is expected to show SOD activity since a UNIPROT search revealed the presence of a SOD homolog (accession numbers: I4Z9K0_9BACT). Indeed, *P. bivia* crude extract (0.13 mg protein) exhibits SOD activity (Fig. 2d).

**Fig. 2:**
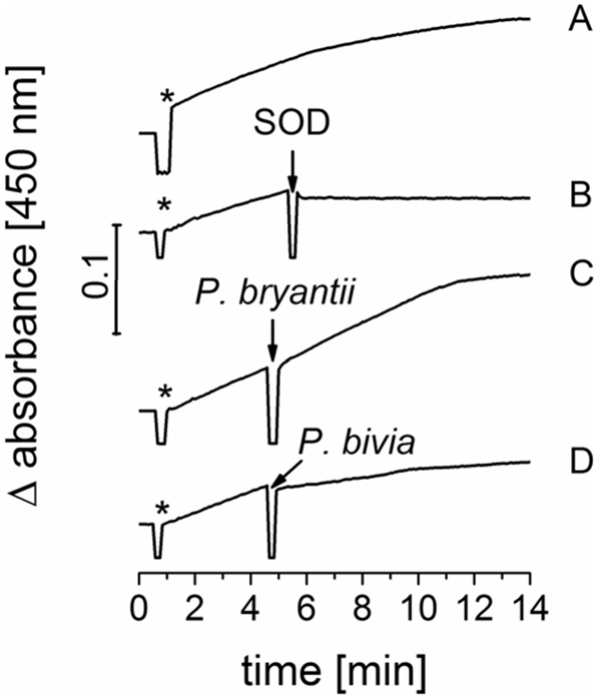
*P. bryantii* cell extracts do not exhibit superoxide dismutase activity. Superoxide dismutase activity was analyzed by monitoring the formation of formazan (450 nm) by superoxide anions produced by xanthine oxidase added at the start of the reaction, indicated by asterisks (*). After ~ 4 min crude extracts (0.13 mg) were added to analyze the SOD activity. **a** no addition. **b** Addition of 100 U bovine SOD. **c** with *P. bryantii* crude extract. **d** with *P. bivia* crude extract

## Electronic supplementary material

Below is the link to the electronic supplementary material.

Supplementary file1 (PDF 143 kb)
