# Supplementary material for: Anoxic cell rupture of Prevotella bryantii by high-pressure homogenization protects the Na+-translocating NADH:quinone oxidoreductase from oxidative damage
Source: Arch Microbiol. 2020 Jan 18;202(5):1263–6. doi: 10.1007/s00203-019-01805-x (PMC7283201; doi:10.1007/s00203-019-01805-x)
Supplement: Supplementary file 1 — Supplementary file1 (PDF 143 kb) [file 203_2019_1805_MOESM1_ESM.pdf]

Archives of Microbiology

Supplementary Data

Anoxic cell rupture of *Prevotella bryantii* by high-pressure homogenization protects the Na^+-translocating NADH:quinone oxidoreductase from oxidative damage

Lena Schleicher[1], Günter Fritz[1] , Jana Seifert[2], Julia Steuber[1*]

[1]Institute of Microbiology, University of Hohenheim, Stuttgart, Germany

[2]Institute of Animal Science, University of Hohenheim, Stuttgart, Germany

In the following, a step-by-step description for anoxic operation of the EmulsiFlex-C3 homogenizer is given. This process is illustrated in fig. S1.

How to prepare the Emulsiflex for anoxic cell rupture (Fig. 1A)

1. Drill three holes (Ø 0.9 cm) into the black plug (Ø 6.4 cm at the top, Ø 5.4 cm at the bottom) which replaces the screw cap of the funnel
2. Insert three 2 mL plastic disposable syringes into the black plug
3. Remove stamps of plastic syringes and close two of the cylinders with red rubber plugs
4. Seal the black plug tight to the funnel with cable ties
5. Connect the nitrogen gas bottle to the first syringe cylinder with the help of a cannula inserted into the red rubber plug
6. Connect the open syringe cylinder with a tubing, ending up in a beaker with water
7. Turn on nitrogen
8. Check for nitrogen bubbles in the beaker and adjust pressure if necessary
9. Flush the funnel (10-15 min) with nitrogen
10. Connect the outlet tubing with a steel cannula (Ø 2 mm, length 6 cm)
11. Switch on the Emulsiflex
12. Switch on the cooling system

Emulsiflex is ready for anoxic cell disruption

How to handle anoxic buffers and suspensions for subsequent cell rupture

1. In the anaerobic chamber, anoxic buffers and cell suspension are ready for cell rupture
2. 2x 50 mL cell lysis buffer is filled into glass syringes and the cannula is sealed with a rubber stopper
3. Cell suspension is filled into a glass syringe and the cannula is also sealed with a rubber stopper
4. Filled syringes are taken out of the anaerobic chamber and placed on ice to cool

Buffers and cells are now ready for the next steps

How to disrupt cells (Fig. 1A, B)

1. Please note that the funnel is flushed with N_2 continuously
2. Inject 50 mL of anoxic cell lysis buffer into the funnel by sticking with the cannula through the red rubber plug on top of the free (third) syringe cylinder.
3. Turn red stop knob of device clockwise and push green knob to start pump
4. Pump anoxic cell lysis buffer through the system for equilibration
5. Press red stop knob to stop the device
6. Inject anoxic cell suspension into the funnel through the red rubber plug of the free (third) syringe cylinder
7. Connect cannula from outlet tubing with the free (third) syringe cylinder
8. Start pump
9. Let the cell suspension run through the tubing back to the funnel (see Fig. 1B)
10. Turn on air pressure. Air pressure at 40 psi, gauge pressure at ≤ 20.000 psi depending on organism

11. To disrupt *P. bryantii* cells maximum pressure (20.000 psi) is applied for 10 min

How to collect broken cells (Fig. 1C)

1. Press red stop knob to stop the device
2. Turn off air pressure
3. Remove the outlet tubing from the funnel and connect it by a cannula to a gas-tight serum bottle. This serum bottle was sealed with a rubber cap fastened with an aluminium ring, and was flushed with N_2. Insert an open cannula into the plug while filling the bottle with crude extract to avoid overpressure.
4. Start pump
5. Pump the disrupted cells out of the device into the gas tight serum bottle
6. Remove open cannula
7. The crude cell extract in the gas-tight serum bottle is placed into the anaerobic chamber for subsequent fractionation and characterization

Cleaning of Emulsiflex

1. Black plug with syringe cylinders is dismantled and cleaned first with 80 % ethanol, then with deionized water
2. All cannulas and tubings are removed and cleaned first with 80 % ethanol, then with deionized water
3. EmulsiFlex is flushed with deionized water, 500 mM NaOH, deionized water and finally with 80 % ethanol
4. Leave 1/3 of a funnel volume of 80 % ethanol in the funnel for storage

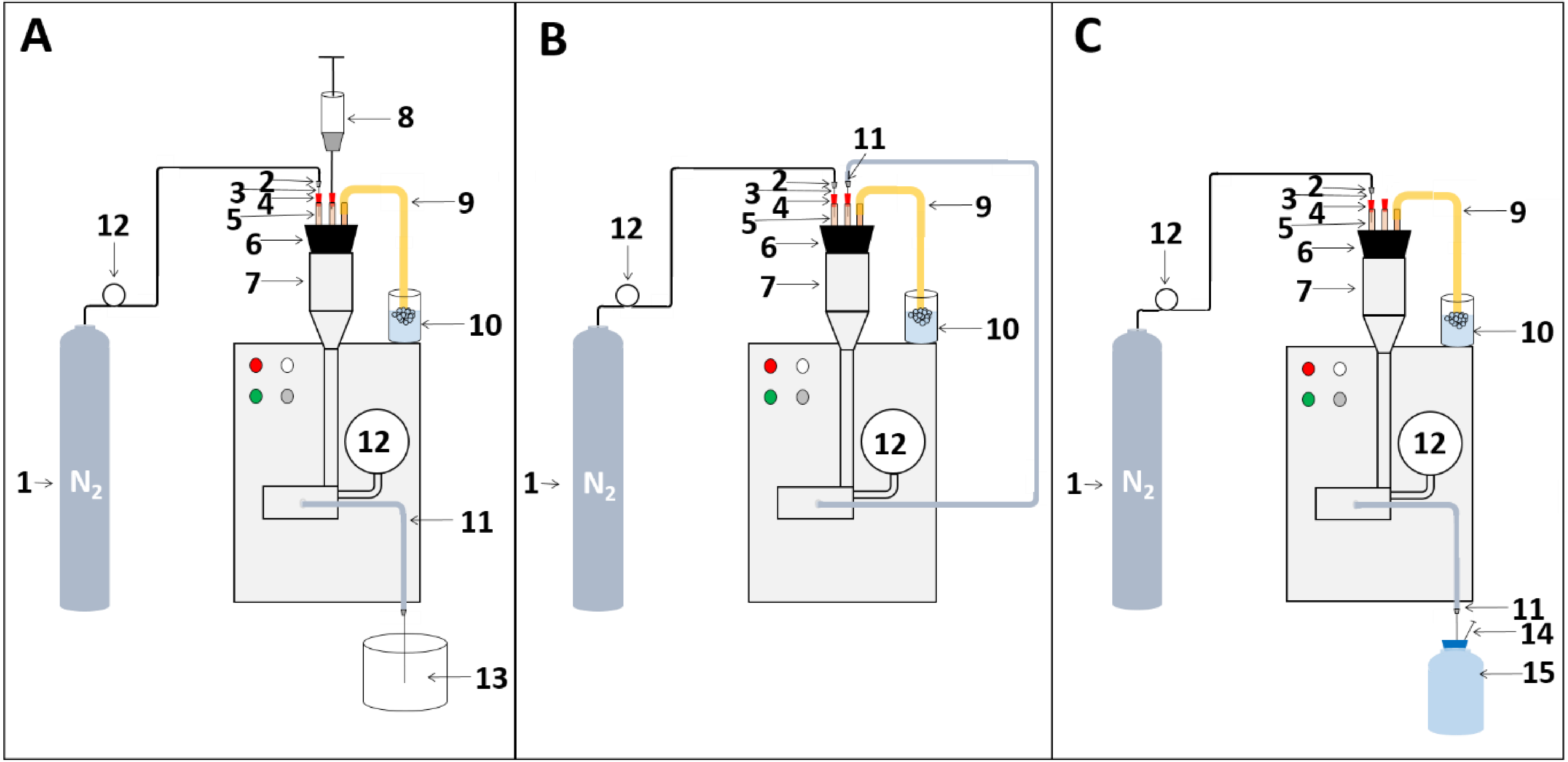

Supplementary figure S1: Anoxic cell disruption with the Emulsiflex. Throughout the process, the N 2 gas bottle (1) is connected to the funnel, which is continuously flushed with N 2. A) Assembly for equilibration of the device with anoxic cell lysis buffer. The sample outlet tubing (11) is connected to the waste (13). B) Assembly for cell disruption. The sample outlet tubing (11) is connected to a syringe cylinder (5) to run the sample through the device and back to the funnel. C) Collection of the disrupted cells. The sample outlet tubing (11) is connected to an anoxic gas-tight serum bottle (15). 1: nitrogen gas bottle, 2: adapter for tubing and cannula, 3: steel cannula, 4: red rubber plug, 5: syringe cylinder (outer part of syringe, without stamp), 6: black plug, 7: funnel (sample container), 8: syringe with buffer or cell suspension, 9: outlet tubing for nitrogen, 10: beaker with water, 11: outlet tubing for broken cells, 12: pressure gauge, 13: waste, 14: open cannula, 15: serum bottle filled with N_2.
